# Sargachromenol Isolated from *Sargassum horneri* Attenuates Glutamate-Induced Neuronal Cell Death and Oxidative Stress through Inhibition of MAPK/NF-κB and Activation of Nrf2/HO-1 Signaling Pathway

**DOI:** 10.3390/md20110710

**Published:** 2022-11-12

**Authors:** Eui-Jeong Han, Chunying Zhang, Hyun-Soo Kim, Ji-Yul Kim, Sang-Muyn Park, Won-Kyo Jung, Ginnae Ahn, Seon-Heui Cha

**Affiliations:** 1 Department of Marine Bio-Food Sciences, Chonnam National University, Yeosu 59626, Korea; 2 Department of Marine Bio and Medical Sciences, Hanseo University, Seosan-si 32158, Korea; 3 National Marine Biodiversity Institute of Korea, Seocheon-kun 33662, Korea; 4 Department of Pharmacology, Ajou University School of Medicine, Suwon 16499, Korea; 5 Research Center for Marine Integrated Bionics Technology and Marine Integrated Biomedical Technology Center, Pukyong National University, Busan 48513, Korea; 6 Department of Biomedical Engineering, New Senior Healthcare Innovation Center (BK21 Plus), Pukyong National University, Busan 48513, Korea

**Keywords:** sargachromenol, *Sargassum horneri*, Neuroprotective effects, oxidative stress, neurons cell death, glutamate

## Abstract

Oxidative stress-induced neuronal cell loss is considered to be the major mechanism underlying the pathogenesis of neurodegenerative diseases, which could be induced by a high concentration of glutamate. In this study, sargachromenol (SC) was isolated from a marine brown seaweed *Sargassum horneri* (*S. horneri*) and its neuroprotective effects against glutamate-induced oxidative stress in HT22 cells were investigated. An MTT assay was applied to assess the cytotoxicity of the SC, and the efficacies of SC were determined by flow cytometry, an analysis of ROS production, quantitative Real-Time PCR, and the Western blot assay. Our results showed that the pretreatment of SC reduced glutamate-induced apoptosis in HT22 cells via inhibiting the sub-G_1_ population, DNA fragmentation, and nuclear condensation, as well as up-regulating anti-apoptotic protein (Bcl-2) and down-regulating apoptotic proteins (Bax, p53, cleaved-PARP, caspase-3, caspase-9, and cytochrome c). Additionally, SC attenuated glutamate-induced oxidative stress by suppressing mitogen-activated protein kinases (MAPKs;ERK, JNK, and p38) and nuclear factor kappa-light-chain-enhancer of activated B cells (NF-κB) signaling (IκBα and NF-κB p65), while activating nuclear factor erythroid-2-related factor 2 (Nrf2)/heme oxygenase 1 (HO-1) signaling (Nrf2; HO-1, and NQO-1). Our results suggest that SC could be used as a pharmacological candidate for the prevention and treatment of neurodegenerative diseases.

## 1. Introduction

Neurodegenerative diseases are disorders caused by neurodegeneration that occur in the central nervous system (CNS) through hallmarks associated with the loss of neuronal structure(s) and function(s) [1]. Glutamate is a major endogenous excitatory neurotransmitter in the vertebrate CNS, which is potentially involved in the pathogenesis of various CNS diseases, whether due to reduced uptake, excessive release, or changes in receptor function [2,3]. Glutamate excitotoxicity is associated with various neurodegenerative diseases, such as Alzheimer’s disease (AD), Parkinson’s disease (PD), cerebral ischemia, epilepsy, multiple sclerosis (MS), and amyotrophic lateral sclerosis (ALS) [4,5]. Glutamate-induced neuronal damage occurs through two main mechanisms. The well-known pathways are mediated by the glutamate receptor N-methyl-D-aspartate (NMDA), which leads to an excessive influx of Ca^2+^, resulting in the activation of calpain and other proteases that mediate cytoskeletal damage, accompanied by mitochondrially derived reactive oxygen species (ROS) and subsequent neuronal cell death [6,7,8]. Another pathway is the inhibition of the influx of the amino acid cystine through the glutamate/cystine antiporter without passing through the glutamate receptor, and reducing the concentration of glutathione, the precursor of cystine in the cell, which increases ROS production, leading to cell death [9,10]. Both mechanisms induce apoptosis by the overexpression of ROS.

In recent years, natural products have gained more attention as alternative or comprehensive therapeutic agents for various neurodegenerative diseases, including seaweed, which has abundant bioactive properties and pharmaceutical value [11,12,13,14]. *Sargassum horneri* (*S. horneri*), a brown seaweed, has been used as a medicinal plant in the treatment of several diseases, such as hypertension, hyperlipidemia, heart disease, and inflammatory diseases (furuncle), and has potential functions in the prevention of osteoporosis [15,16,17]. In addition, *S. horneri* is considered to be a rich source of bioactive compounds such as sulfated polysaccharides, fucoxanthin, proteoglycan, phlorotannins, polyphenol, chromanols, and sargachromenol (SC) [15,16,17,18,19,20].

Among them, *S. horneri*-derived SC can regulate the transcription of AP-1 in dermal fibroblasts and can protect UV-A-induced cell damage [20]. Likewise, the SC isolated from *Sargassum macrocarpum* (*S. macrocarpum*) was reported to work like a brain-derived neurotrophic factor (BDNF), which is a key molecule for memory in healthy and pathological brains used to enhance nerve growth factor-dependent neurite outgrowth that promotes activity in PC12 cells [19]. However, studies regarding the effects of SC from *S. horneri* on neuro-diseases have not been investigated. Therefore, this study aims to demonstrate the antioxidative mechanism underlying the neuroprotective effects of SC, a plastoquinone compound isolated from *S. horneri*, against glutamate-induced toxicity in HT22 cells.

## 2. Results

### 2.1. SC Attenuates Glutamate-Induced Cell Death in Neuronal HT22 Cells

**Figure 1:**
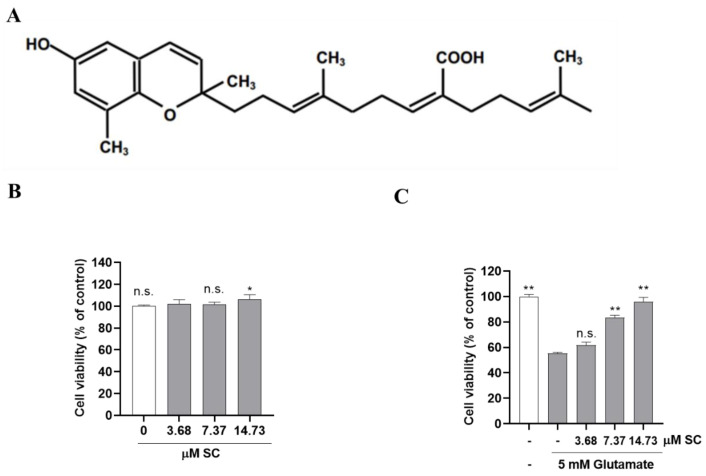
SC prevents glutamate-induced toxicity in HT22 cells. (**A**) Chemical structure of SC. (**B**) HT22 cells were incubated with the indicated concentrations of SC for 24 h. The statistical symbols (* *p* < 0.05; n.s., no significance) indicate significant differences from the 3.68 μM SC-treated group. (**C**) HT22 cells were incubated with or without indicated concentration of SC for 1 h, and then further incubated with 5 mM glutamate for 24 h. Cell viability was measured by MTT assay. Experiments were performed in triplicate. The statistical symbols (* *p* < 0.05; ** *p* < 0.01; n.s., no significance) indicate significant differences from the glutamate-treated group.

To determine whether sargachromenol (SC, Figure 1A, Supplementary Figure S1) protects against glutamate-induced toxicity, HT22 cells were treated with either various concentrations of SC or glutamate alone or were preincubated with SC for 1 h and then further incubated with glutamate. SC alone showed no toxicity toward HT22 cells in the concentration range tested (3.68–14.73 µM, Figure 1B). As shown in Figure 1C, the cell viability treated with glutamate was significantly decreased to 55.39 ± 0.42% compared to the non-treated control cells. Interestingly, compared with the presence of 5 mM of glutamate alone, the pretreatment with SC in the co-treated group increased cell viability; in particular, 14.73 µM of SC in the co-treated group increased cell viability (93.22 ± 1.75%) similar to that of the control (Figure 1C), indicating that SC has cytoprotective effects against glutamate-induced damage in HT22 cells.

### 2.2. SC Inhibits Cellular Damage by Modulating the Expression of Apoptosis-Mediated Proteins

**Figure 2:**
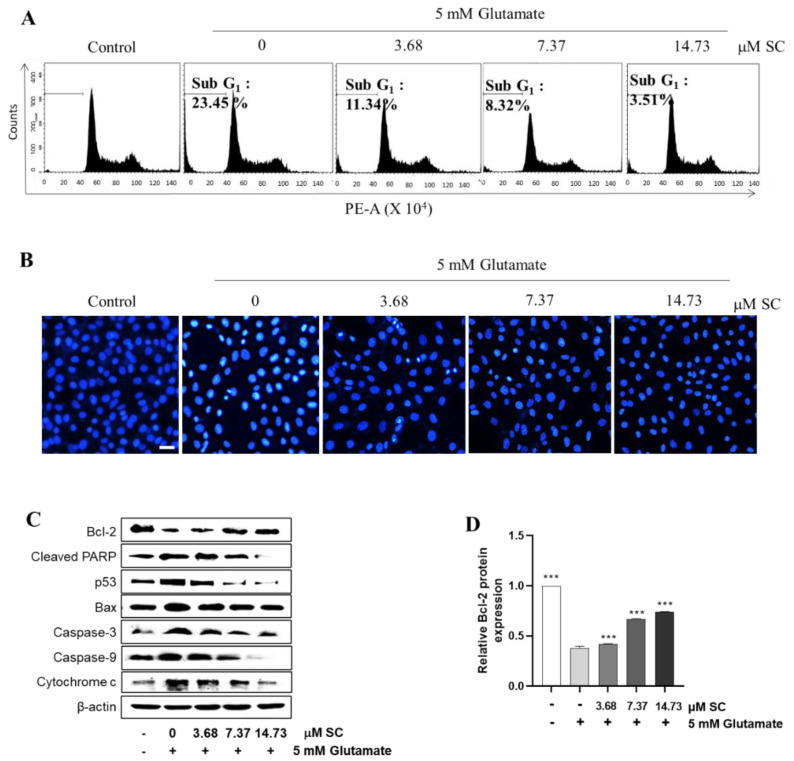

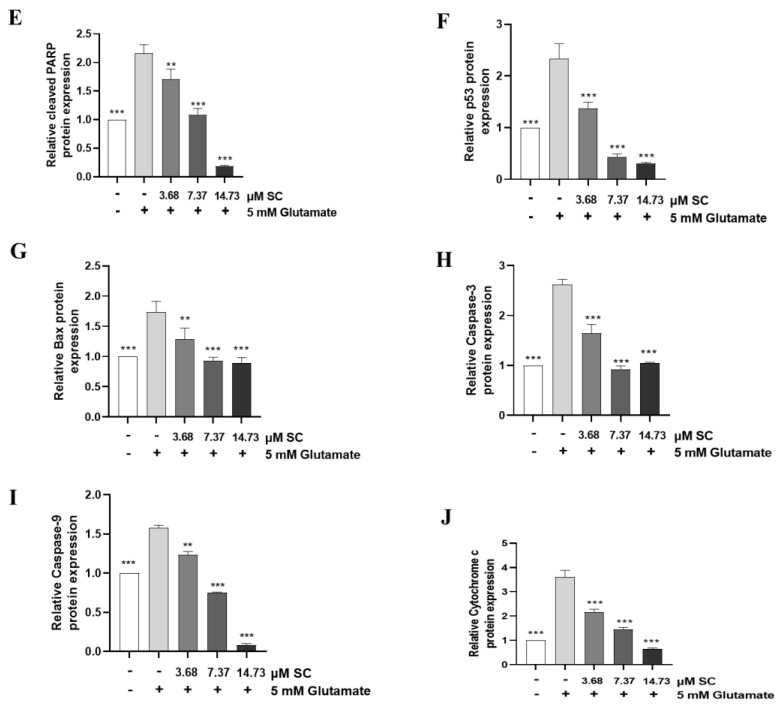
SC protects against the damage caused by glutamate in HT22 cells. The cells were incubated with or without indicated concentration of SC for 1 h, and then further incubated with 5 mM glutamate for 12 h. (**A**) Effect of SC on sub-G_1_ DNA content in glutamate-treated HT22 cells. The sub-G_1_ DNA content was observed by PI staining. (**B**) Effects of SC on glutamate-induced apoptotic bodies were determined by Hoechst 33342 staining. Scale bar 100 μm (**C**–**J**) Effects of SC on protein expression related to apoptosis were observed by Western blot assay. The statistical symbols (** *p* < 0.01; *** *p* < 0.001) indicate significant differences from the glutamate-treated group.

In order to confirm whether SC showed cytoprotective effects against glutamate-induced cell death, we examined the production of sub-G1 DNA contents, nuclear condensation/fragmentation, and expression of apoptosis-related proteins expression [21]. Thus, we first checked the DNA content using flow cytometry. As shown in Figure 2A, compared with the non-treated control cells (1.21%), the glutamate-treated cells significantly induced the sub-G_1_ population (23.45%), whereas the pretreatment of SC prior to glutamate’s addition decreased the sub-G_1_ population up to 3.51% at 14.76 µM per dose. Next, we confirmed the morphological changes, as shown in Figure 2B, caused by glutamate. The nuclear staining in Figure 2B indicated that the glutamate-treated cells exhibited a greater degree of fragmentation and nuclear condensation, which are characteristics of apoptosis, compared to the non-treated cells (control). However, these processes were effectively reduced by the pretreatment with SC (Figure 2B). Further, we checked the expression levels of apoptosis-mediated proteins. The SC pretreatment markedly increased the expression of Bcl-2 protein that was decreased by glutamate (Figure 2C,D). In contrast, the expression levels of pro-apoptotic proteins (Bax, p53, and cleaved-PARP) increased by glutamate were down-regulated by the pretreatment of SC (Figure 2C,E–G). Moreover, the pretreatment with SC decreased the expression levels of the apoptosis sub-molecules, caspase-3 and caspase-9 (Figure 2C,H,I), and finally inhibited the expression of cytochrome c (Figure 2C,J), suggesting that SC protects HT22 cells against glutamate by inhibiting apoptosis.

### 2.3. SC Reduces the Production of Intracellular ROS via Inactivation of the Mitogen-Activated Protein Kinases (MAPK) Signaling Pathway

**Figure 3:**
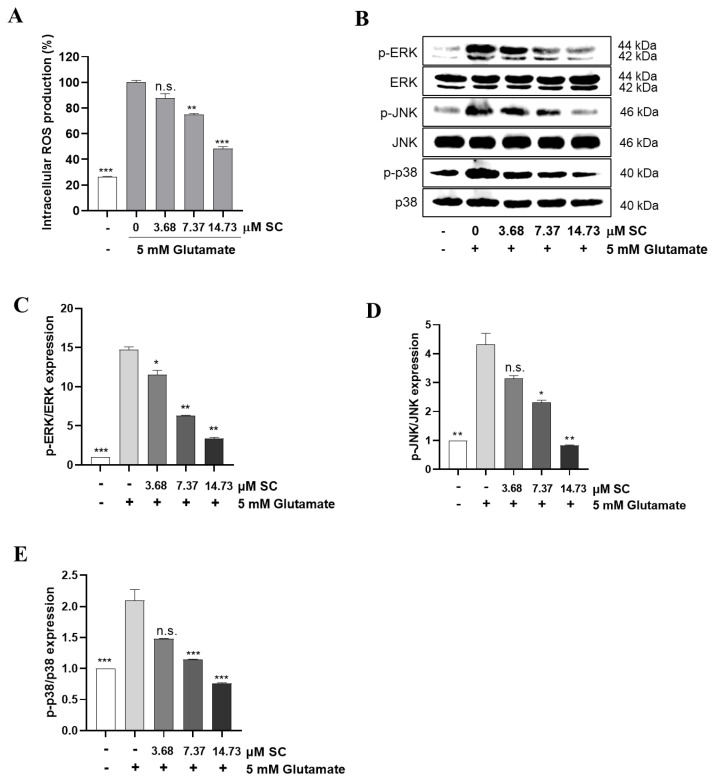
SC reduces the intracellular ROS production and inactivates the MAPKs in glutamate-treated HT22 cells. The cells were incubated with or without indicated concentration of SC for 1 h, and then further incubated with 5 mM glutamate for 12 h. (**A**) Effect of SC on intracellular ROS production. (**B**–**E**) Effect of SC on protein expression of MAPKs. The statistical symbols (* *p* < 0.05; ** *p* < 0.01; *** *p* < 0.001; n.s., no significance) indicate significant differences from the glutamate-treated group.

Cytochrome *c* is an interspace protein between the outer and inner membranes of the mitochondria, which is a key organelle that controls oxidative stress balance to preserve cells [22]. We found that the cytochrome *c* release caused by glutamate was reduced by the SC treatment prior to the glutamate treatment (Figure 2C,J). So, to confirm whether SC attenuates the overproduction of ROS—a representative indicator of oxidative stress—caused by glutamate, we performed a DCFH-DA assay. As shown in Figure 3A, the intracellular rate of ROS production significantly increased compared to the non-treated cells in the glutamate-treated cells, whereas the number of ROS were significantly reduced by the SC-pretreated cells. Interestingly, SC also reduced the phosphorylation of ERK, JNK, and p38, which are sub signals of oxidative stress (Figure 3B–E). According to these results, we confirmed that SC significantly suppressed the oxidative stress induced by glutamate by inhibiting the activation of MAPKs.

### 2.4. SC Inhibits the Activation of Nuclear Factor Kappa-Light-Chain-Enhancer of Activated B Cells (NF-κB) Signaling in Glutamate-Treated HT22 Cells

**Figure 4:**
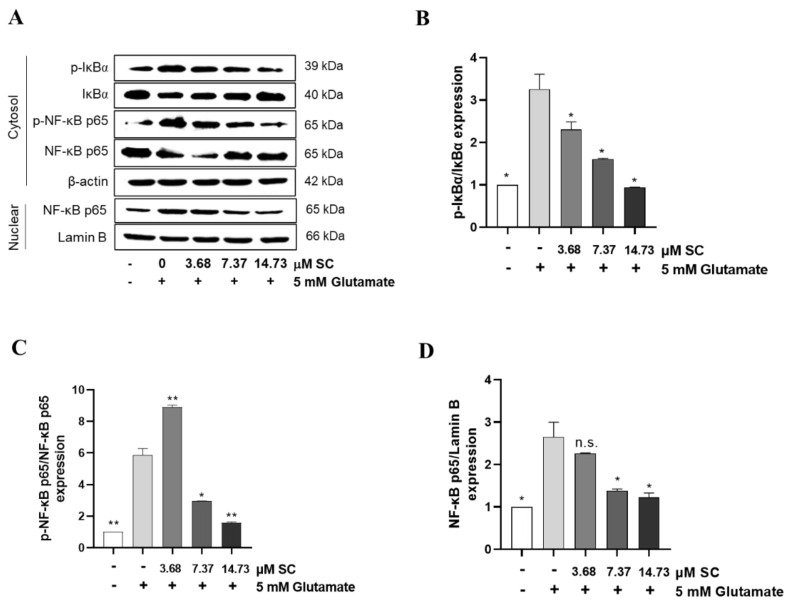
SC inhibits the activation of NF-κB signaling pathway in glutamate-treated HT22 cells. HT22 cells were incubated with various doses of SC for 1 h, and then further incubated with 5 mM glutamate for 1 h. The expression levels of proteins involved in NF-κB signaling pathway such as IκBα (**A**,**B**), cytoplasmic NF-κB p65 (**A**,**C**), and nuclear NF-κB p65 (**A**,**D**) were detected by Western blot assay. The statistical symbols (* *p* < 0.05; ** *p* < 0.01; n.s., no significance) indicate significant differences from the glutamate-treated group.

To determine whether SC could attenuate the glutamate-induced activation NF-κB signaling, we ascertained the degree of NF-κB phosphorylation using the Western blot assay. As shown in Figure 4, the glutamate-treated cells markedly increased the phosphorylation of IκBα and NF-κB p65 in the cytosol as well as the nuclear translocation of NF-κB p65 into the nucleus compared to the non-treated cells. However, the phosphorylation of IκBα and the shift of NF-κB p65 to the nucleus induced by glutamate were nullified in a dose-dependent manner by the pretreatment of SC (Figure 4A,D).

### 2.5. SC Activates Nuclear Factor Erythroid-2-Related Factor 2 (Nrf2)/Heme Oxygenase 1 (HO-1) Signaling Pathway in Glutamate-Treated HT22 Cells

**Figure 5:**
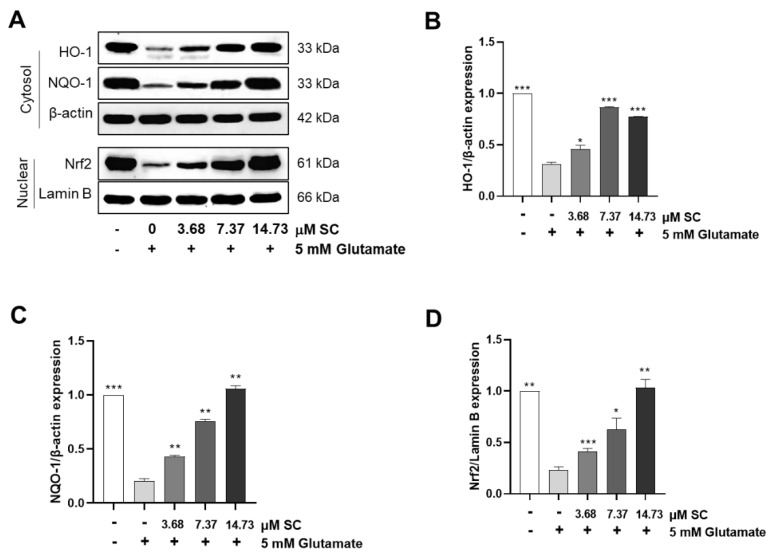
SC activates Nrf2/HO-1 signaling pathway in glutamate-treated HT22 cells. The cells were incubated with various doses of SC for 1 h, and then further incubated with 5 mM glutamate for 12 h. The expression levels of proteins involved in Nrf2/HO-1 signaling pathway such as HO-1 (**A**,**B**), cytoplasmic NQO-1 (**A**,**C**), and Nrf2 (**A**,**D**) were detected by Western blot assay. The statistical symbols (* *p* < 0.05; ** *p* < 0.01; *** *p* < 0.001) indicate significant differences from the glutamate-treated group.

Nrf2, a master transcription factor involved in antioxidant signaling and the cell survival response, regulates a wide battery of cytoprotective responses and helps to attenuate neurodegenerative diseases [23]. Furthermore, HO-1 is a crucial antioxidant enzyme, and its induction was shown to be induced by Nrf2 activation; however, the expression and involvement of the Nrf-2/HO-1 antioxidant axis in the protective effect provided by SC against glutamate-induced HT22 cellular damage has not yet been studied. Furthermore, Nrf2/HO-1 signaling can affect to the inhibition of NF-κB activation and nuclear translocation [24]. Therefore, to determine whether SC affects the Nrf2/HO-1 signaling pathway, we assessed Nrf2/HO-1 protein expression using the Western blot assay. The immunoblotting results demonstrated that the cells administered the glutamate treatment decreased the protein expression of Nrf2 in the nuclear (Figure 5A,D) and HO-1 in the cytosol compared to non-treated cells, for which the reduction rates were recoded as 71.03% (Figure 5A,B,D). In contrast, the expression of Nrf2 and HO-1 protein increased with the pretreatment of SC in a dose-dependent manner. The Nrf2 expression induction rates were recorded to be around 18% at 3.68 μM SC, and 44% and 39% at 7.37 μM and 14.73 μM, respectively, in comparison with the glutamate-treated cells (Figure 5A,B,D).

NAD(P)H quinine oxidoreductase-1 (NQO-1) is a cytosolic enzyme that protects cells against chemically induced oxidative stress, and the gene is regulated by Nrf2. So, the protein expression of NQO-1 was determined to confirm whether Nrf2 is activated by SC. As shown in the Figure 5A,C the protein level of NQO-1 was decreased by the glutamate treatment, whereas the reduction was preserved by the treatment of SC in a dose-dependent manner.

**Figure 6:**
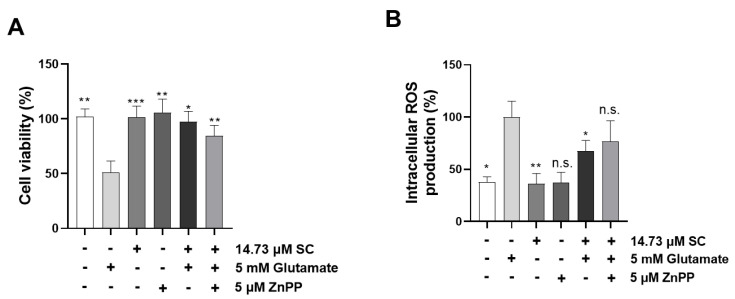
Effect of HO-1 inhibitor ZnPP on cell viability and intracellular ROS levels in SC-pretreated HT22 cells under glutamate treatment. The HT22 cells were incubated with 5 μM ZnPP for 1 h with or without SC (14.73 µM) followed by 5 mM glutamate treatment for 1 h. Then, cell viability (**A**) was determined with MTT assay and intracellular ROS levels (**B**) were measured by DCFH-DA assays 24 h after exposure to glutamate. The value of cell viability was set to 100% for the non-treated control cells, and the intracellular degree of ROS production was set to 100% for the glutamate-treated cells. Values are expressed as the mean ± SE calculated from three replicates. The statistical symbols (* *p* < 0.05; ** *p* < 0.01; *** *p* < 0.001; n.s., no significance) indicate significant differences from the glutamate-treated group.

**Figure 7:**
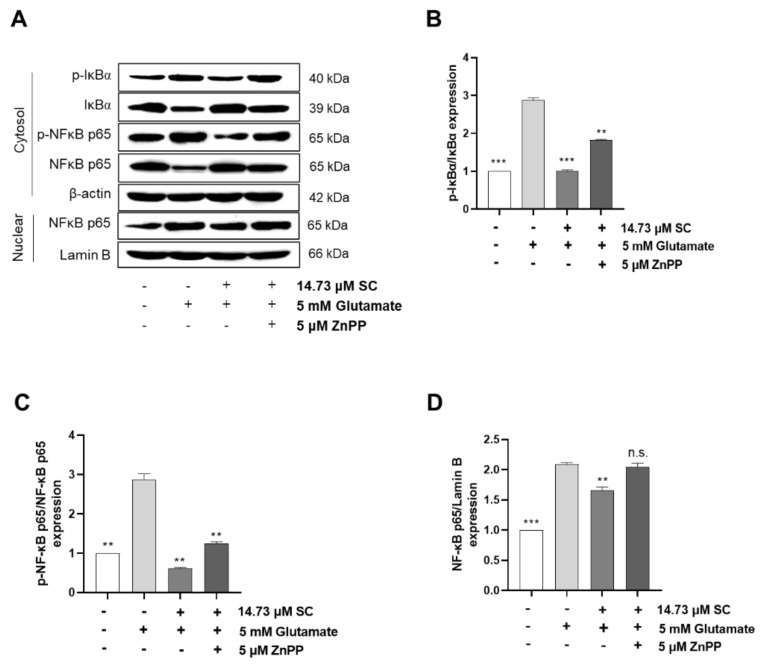
Effects of SC on the NF-κB signaling pathway in HT22 cells with HO-1 inhibited. Cells were treated with 5 μM ZnPP for 1 h with SC (14.73 µM) followed by 5 mM glutamate for 1 h. Subsequently, phosphorylation levels of IκBα (**A**,**B**) cytoplasmic NF-κB p65 (**A**,**C**), and nuclear NF-κB p65 (**A**,**D**) involved in the NF-κB signaling pathway were detected by Western blot assay. Values are expressed as the mean ± SE calculated from three replicates. The statistical symbols (** *p* < 0.01; *** *p* < 0.001; n.s., no significance) indicate significant differences from the glutamate-treated group.

To further confirm the cytoprotective effects of SC on the Nrf2/HO-1 signaling pathway, the cells were treated with the HO-1 inhibitor ZnPP with or without SC and glutamate. The results showed that the additional ZnPP significantly reduced the cell viability compared with the SC-pretreated cells induced by glutamate alone (Figure 6A). Moreover, the degree of intracellular ROS production was significantly increased by adding ZnPP to the cells that were co-treated with SC and glutamate (Figure 6B). These results indicated that the supplementation of ZnPP can clearly inhibit the activation of the Nrf2/HO-1 signaling pathway. Subsequently, we further investigated whether the additional ZnPP plays a role in the NF-κB signaling pathway. The results showed that the addition of ZnPP promoted the phosphorylation of IκBα and NF-κB p65 in the cytosol and the translocation of NF-κB p65 into the nucleus compared to the SC and glutamate co-treated cells (Figure 7A–D); these results demonstrated that the inactivation of the HO-1 pathway was not conducive to the cytoprotective effect of SC, which is mainly reflected in the restoration of the NF-κB signaling pathway through the addition of ZnPP.

## 3. Discussion

Almost all the brains of the elderly show characteristic changes related to neurodegeneration [25]. With the trend of an increasingly elderly and longer-living population, the cognitive and memory impairments caused by neurodegenerative diseases have become a significant public health problem worldwide [26]. The loss of neuronal cells caused by oxidative stress is considered to be a potential major issue in the pathogenesis of neurodegenerative diseases [27]. Therefore, the extensive development of neuroprotective active ingredients is of great significance for the prevention and treatment of neurodegenerative diseases. In the present study, a higher concentration (14.73 µM) of the marine active compound SC significantly increased cell viability and inhibited glutamate-induced damage in HT22 neural cells (Figure 1). These results implying that the potential beneficial effects of SC towards conserving the neuroprotective properties provided by HT22 cells.

Glutamate-induced oxidative stress results in apoptosis and necrosis, which are considered to be the two main death pathways of neurons in neurodegenerative diseases [28,29,30]. According to our results, the DNA fragmentation and nuclear condensation elicited by glutamate was decreased due to the pretreatment with SC, and the flow cytometry data provided evidence of the ability of SC to attenuate apoptosis caused by the reduction of sub-G1 populations in the cell cycle (Figure 2A,B), indicating SC’s protective effects against glutamate-induced apoptosis in HT22 cells. In addition, the apoptotic pathway occurs through the activation of p53 and pro-apoptotic proteins (e.g., Bax, Bik, and Bmf) in the cytoplasm, thereby inhibiting the anti-apoptotic proteins (e.g., Bcl-2, Mcl-1, and Bcl-xL). This in turn leads to apoptosis-promoting proteins such as cytochrome C and activates the main candidate of caspase-9, which causes a series of reactions to activate caspases-3, -6, and -7 [31]. During this series of apoptosis processes, the phenomena of the biochemical and morphological modifications of key regulatory molecules increases, such as that of PARP, which is responsible for DNA repair, transcription, and stability [32]. In the present study, the glutamate treatment down-regulated the expression of Bcl-2 and up-regulated the expression of pro-apoptotic proteins (Bax, p53 and cleaved-PARP), apoptosis sub-molecules (caspase-3 and caspase-9), and cytochrome *c*, while the SC pretreatment reversed those effects induced by glutamate (Figure 2C). These results suggest that SC contributed to preventing glutamate-induced normocellular apoptosis by its favorable regulation of apoptosis-mediated protein expression. The family of MAPKs, including ERK, JNK, and p38 kinases, play an important role in signal transduction from the plasma membrane to the nucleus and their activation is associated with cell survival and death in many neurodegenerative diseases [33,34]. Previous studies indicated that treatment with an inhibitor of ERK can prevent the glutamate-induced phosphorylation of ERK in HT22 cells and primary cortical neurons [35]. JNK and p38 kinase were activated in the process of HT22 cell death induced by glutamate [30,36]. In accordance with our results, the phosphorylation of ERK, JNK, and p38 caused by glutamate was rescued by the SC treatment (Figure 3). These results suggest that the inhibition of MAPKs is a key component of the SC-mediated neuroprotection against glutamate-induced cell death in HT22 cells. In addition, the NF-κB signaling pathway is involved in the regulation of target genes in the CNS involved in controlling physiological functions and pathological processes related to neurodegeneration [37,38,39]. Under inactive conditions, NF-κB is present as a three-subunit complex, with the prototypical components being p50, p65, and IκBα. Once activated, IκBα is degraded in the proteasome, and the NF-κB dimer releases and translocates to the nucleus to bind with promoter regions of the NF-κB responsive genes, thus up-regulating the expression of pro-apoptotic factors in neurons [40,41]. Accumulating evidence indicates that activated NF-κB/p65 is closely associated with neuronal programmed death, which is reversed by inhibiting the activation of NF-κB/p65 [42,43,44,45]. Consistent with these reports, this study proved that the inhibition of the NF-κB/p65 signaling pathway by SC contributed to its protective effect on HT22 cells (Figure 4). On the contrary, neuronal cells activate an endogenous antioxidant defense system against oxidative stress, especially Nrf2, which plays an essential role in protecting cells from oxidative damage by promoting the expression of antioxidant enzymes such as HO-1, NQO-1, and GCLC [46]. In the neurodegenerative brain, in which the neurodegeneration derives from oxidative stress, the amount of located Nrf2 in the nucleus is less than that in the cytoplasm, which is accompanied by inactivated antioxidant enzymes [47]. A previous study showed that an ethanol extract of *S. serratifolium* inhibits RANKL-induced oxidative stress by the activation of the Nrf2/HO-1 signaling pathway [32]. In this study, the pretreatment of SC attenuated the glutamate-induced down-regulation of Nrf2 in the nucleus as well as HO-1 and NQO-1 in the cytosol (Figure 5). The blockage of the Nrf2/HO-1 signaling pathway not only resulted in a significant increase in the level of intracellular ROS (Figure 6) but also attenuated the inhibitory effect of SC on the NF-κB signaling pathway (Figure 7). These results suggest that the Nrf2/HO-1 signaling pathway indispensably contributes to SC’s protection against glutamate-induced oxidative stress in HT22 cells.

## 4. Materials and Methods

### 4.1. Chemicals

Dulbeco’s modified Eagle’s medium (DMEM), fetal bovine serum (FBS), and penicillin/streptomycin were purchased from Gibco BRL (Paisley, UK). 3-(4-,5-dimethyl thiazol-2-yl)-2-,5-diphynyltetrasolium diphenyltetrazolium bromide (MTT), dimethyl sulfoxide (DMSO), 2′,7′-dichlorodihydroflurescin diacetate (DCFH-DA), L-glutamate (glutamate), Hoechst 33342, and propidium iodide (PI) reagent were obtained from Sigma Aldrich Chemical Co. (St. Louis, MO, USA). Primary and secondary antibodies were purchased from Cell Signaling Technology Inc. (Beverly, MA, USA). The other chemicals and reagents used were of the highest grade available commercially. Sargachromenol (SC) isolated from *S. horneri* was extracted with 70% ethanol and partitioned with *n*-hexane and ethyl acetate, respectively. The hexane fraction was fractionated using ODS open column chromatography via hexane/ethyl acetate step-gradient elution. The best fraction was subjected to further purification using Prep HPLC employing a semipreparative C18 (Cosmosil, 10 µm, and 10 × 250 mm) column on a YL900 HPLC system (Young Lin, UK) [48]. The chemical structure as well as 1H and 13C NMR spectra of SC are shown in Figure 1A and Supplementary Figure S1, respectively.

### 4.2. Cell Culture

A murine hippocampal cell line, HT22 cells, were purchased from Merck Millipore (Darmstadt, Germany). In addition, it was grown in DMEM supplemented with 10% FBS and 1% penicillin/streptomycin at 37 °C under 5% CO_2_ incubator.

### 4.3. Cell Viability Assay

To measure cell viability of SC, MTT assay was conducted. HT22 cells were plated on 96-well plate at a density of 1 × 10^4^/well and incubated for stabilization. The cells were incubated for 24 h with the various concentrations of SC (3.68, 7.37, and 14.73 µM) and treated for 4 h with the addition of 15 µL MTT solution (5 mg/mL). The stained cells were dissolved with the 100 µL DMSO reagent, and the absorbance at 570 nm was measured using an ELISA (enzyme-linked immune sorbent assay) reader (Sunrise, Tecan Co., Ltd., Grödig, Austria).

In addition, to evaluate the cell-protective effects of SC on glutamate-induced cytotoxicity in HT22 cells, MTT assay was performed following the same procedure as above. Briefly, the cells were incubated with non-cytotoxic dose of SC (3.68, 7.37, and 14.73 µM) for 1 h and then stressed by 5 mM glutamate. After 24 h, cells were treated using MTT reagent and absorbance was measured at the same wavelength.

### 4.4. Measurement of Sub-G1 Hypodipolid Cells

To determine the effect of SC on increased sub-G1 content via glutamate treatment, HT22 cells were plated on 6-well plate at a density of 5 × 10^5^/well and PI staining was performed. The cells were incubated with SC (3.68, 7.37, and 14.73 µM) for 1 h and stressed by glutamate for 12 h. After incubation, the cells were stained by 500 µL of 2 mM PBS-EDTA containing PI reagent (50 µg/mL) and RNase A (0.2 µg/mL) (Promega, WI, USA). Stained cells were measured using a CytoFLEX flow cytometer (Beckman Coulter, Brea, CA, USA).

### 4.5. Measurement of Apoptotic Body Formation

To identify whether SC suppressed the apoptotic formation of apoptotic bodies increased by glutamate treatment, HT22 cells were seeded on 6-well plate at a density of 5 × 10^5^/well and Hoechst 33,342 staining was performed. The cells were pre-treated with SC (3.68, 7.37, and 14.73 µM), and stressed by glutamate. After 12 h, the cells were stained with Hoechst 33,342 reagent (2 µg/mL) at 37 °C for 30 min. The morphological aspect of stained cells was measured by a fluorescence microscope (Olympus, Shinjuku, Japan).

### 4.6. Measurement of Intracellular ROS Production

Intracellular ROS production was measured using DCFH-DA assay. The cells (1.6 × 10^4^/well) were pretreated with SC (3.68, 7.37, and 14.73 µM) for 1 h prior to glutamate stimulation. After 12 h, cells were treated with DCFH-DA reagent (0.5 mg/mL) and the fluorescence was measured by recording values at excitation 485 nm/emission 525 nm. The intracellular ROS production was represented by comparing percentage of only glutamate-treated cells.

### 4.7. Western Blot Assay

To investigate effects of SC on the expression levels of apoptosis-related molecules, proteins related to inflammatorily mediated pathways—such as MAPKs, NF-κB, and Nrf2/HO-1 pathway—were determined by Western blot assay. The cells were extracted using nuclear and cytoplasmic extraction kit (Thermo, Rockford, IL, USA). The obtained cytoplasmic (40 μg) and nuclear proteins (40 μg) were electrophoresed using SDS-polyacrylamide gel and then transferred to a hybridization nitrocellulose membrane (Merck Millipore, Darmstadt, Germany). The membranes were blocked and incubated with 5% skim milk and subsequently incubated with primary antibodies for Bcl-2 (1:1000 dilution), cleaved PARP (1:1000 dilution), Bax (1:1000 dilution), p53 (1:1000 dilution), caspase caspase-3 (1:1000 dilution), caspase-9 (1:1000 dilution), cytochrome C (1:1000 dilution), extracellular signal-regulated protein kinase—ERK (1:1000 dilution), p-ERK (1:1000 dilution), c-Jun N-terminal protein kinase (JNK) (1:1000 dilution), p-JNK (1:1000 dilution), p38 (1:1000 dilution), p-p38 (1:1000 dilution), IkBα (1:1000 dilution), p-IkBα (1:1000 dilution), p65 (1:1000 dilution), p-p65 (1:1000 dilution), HO-1 (1:1000 dilution), Nrf2 (1:1000 dilution), NQO1, Lamin B (1:1000 dilution), and β-actin (1:10,000 dilution). Lamin B and β-actin were used as internal controls for nuclear proteins and cytoplasmic proteins, respectively. The secondary antibodies were the HRP-conjugated anti-mouse IgG and anti-rabbit IgG (1:5000, Cell Signaling Technology Inc. Beverly, MA, USA). The bands were detected using an enhanced Super Signal West Femto Maximum Sensitivity Substrate (Thermo, Burlington, Canada) reagents. To visualize the bands, images were observed using Davinci K ChemiDoc (Young Hwa scientific Co., Ltd., Seoul, Korea) and analyzed using Image J software (US National Institutes of Health, Bethesda, MD, USA).

### 4.8. Statistical Analysis

Data were analyzed using the SPSS package (SPSS, Version 21.0, Chicago, IL, USA). Values were shown as means ± standard error (SE). The mean values of the tail intensity from each treatment were compared using one-way analysis of variance (ANOVA) followed by Duncan’s multiple range test. A *p*-value less than 0.05 was considered significant.

## 5. Conclusions

The present study demonstrated that SC, isolated from *S. horneri*, plays a critical neuroprotective role by protecting HT22 cells against glutamate-induced apoptosis via modulating the expression of apoptosis-mediated proteins, suppressing the MAPK/NF-κB signaling pathway, and activating the Nrf2/HO-1 signaling pathway in oxidative stress-induced neuronal HT22 cells. However, further studies are necessary to verify its neuroprotective mechanism in vivo, such as those involving mice or zebrafish. Our study suggests that SC could be a promising compound for the prevention and treatment of neurodegenerative diseases.

## Data Availability

The data used to support the findings of this study are available from the corresponding author upon request.

## Supplementary Materials

The following supporting information can be downloaded at: https://www.mdpi.com/article/10.3390/md20110710/s1, Figure S1: 1H and 13C NMR spectrum (a) and chemical shift (b) of sargachromenol.

## Abbreviations

AD: Alzheimer’s disease; ALS: amyotrophic lateral sclerosis; ANOVA: one-way analysis of variance; BDNF: brain-derived neurotrophic factor; CNS: central nervous system; DCFH-DA: 2′,7′-dichlorodihydroflurescin diacetate; DMEM: Dulbeco’s modified Eagle’s medium; DMSO: dimethyl sulfoxide; ELISA: enzyme-linked immune-sorbent assay; ERK: extracellular signal regulated protein kinase; FBS: fetal bovine serum; MAPKs: mitogen-activated protein kinases; MS: multiple sclerosis; MTT: 3-(4-,5-dimethyl thiazol-2-yl)-2-,5-diphynyltetrasolium diphenyltetrazolium bromide; NF-κB: nuclear factor-kappa B; NMDA: N-methyl-D-aspartate; NQO-1: oxidoreductase-1; Nrf2/HO-1: nuclear factor erythroid-2-related factor 2/heme oxygenase-1; PD: Parkinson’s disease; PI: propidium iodide; ROS: reactive oxygen species; SC: Sargachromenol; *S. horneri*: *Sargassum horneri*; *S. macrocarpum*: *Sargassum macrocarpum*; JNK: c-Jun N-terminal protein kinase.
