# Supplementary material for: Sargachromenol Isolated from Sargassum horneri Attenuates Glutamate-Induced Neuronal Cell Death and Oxidative Stress through Inhibition of MAPK/NF-κB and Activation of Nrf2/HO-1 Signaling Pathway
Source: Mar Drugs. 2022 Nov 12;20(11):710. doi: 10.3390/md20110710 (PMC9695719; doi:10.3390/md20110710)
Supplement: Supplementary file 1 [file marinedrugs-1969907-supplementary.pdf › marinedrugs-1969907-supplementary.pdf]

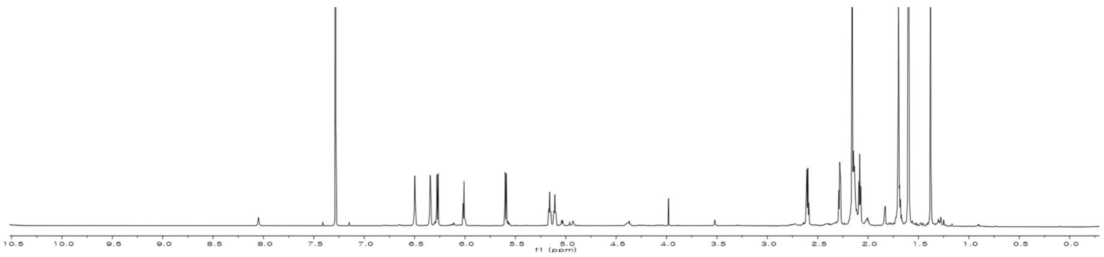

^{1}H-NMR spectrum of sargachromenol ($CDCl_3$, 800 MHz)

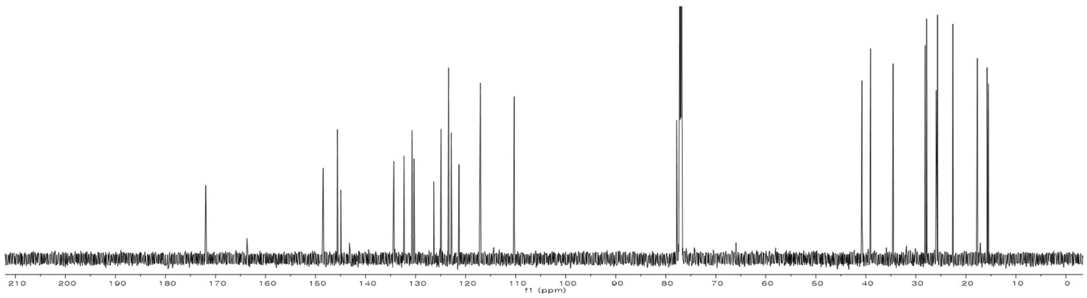

^{13}C-NMR spectrum of sargachromenol ($CDCl_3$, 200 MHz)

(**a**)

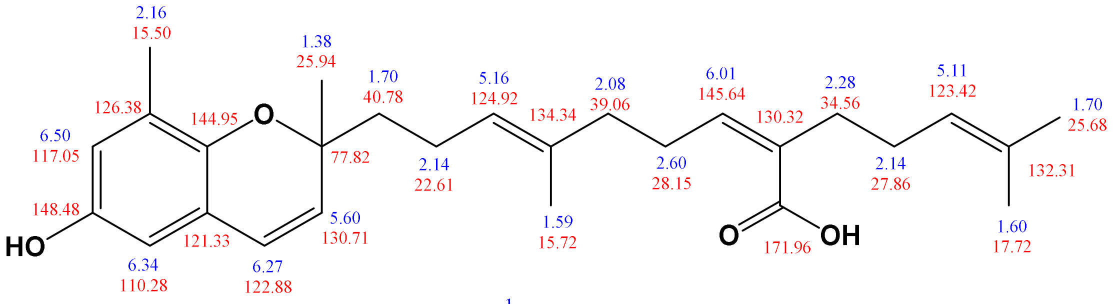

(**b**)

Supplementary figure S1. ^{1}H and ^{13}C NMR spectrum (a) and chemical shift (b) of sargachromen ol. The 1H NMR spectrum of sargachromenol exhibited resonances for two ortho-coupled aromatic proton s (δH 6.27 and 5.60), two meta-coupled aromatic protons (δH 6.50 and 6.34), three olefinic protons (δH 6.0 1, 5.16, and 5.11), six sets of methylene protons (δH 2.60, 2.28, 2.15, 2.14, 2.08, and 1.70), and five methyl pr otons (δH 2.16, 1.70, 1.60, 1.59, and 1.38). The 13C NMR spectrum of sargachromenol showed resonances fo r a carbonyl carbon (δC 171.9), a phenolic carbon (δC 148.4), four aromatic methine carbons (δC 130.7, 122. 8, 117.0, and 110.2), three methinyl vinylic carbons (δC 145.6, 124.9, and 123.4), seven nonprotonated carbo ns (δC 144.9, 134.3, 132.3, 130.7, 130.3 126.3, and 121.3), six methylene carbons (δC 40.7, 39.0, 34.5, 28. 1, 27.8, and 22.6), and five methyl carbons (δC 25.9, 25.6, 17.7, 15.7, and 15.5).
